# Retraction: Murine hypothalamic destruction with vascular cell apoptosis subsequent to combined administration of human papilloma virus vaccine and pertussis toxin

**DOI:** 10.1038/srep46971

**Published:** 2018-05-11

**Authors:** Satoko Aratani, Hidetoshi Fujita, Yoshiyuki Kuroiwa, Chie Usui, Shumpei Yokota, Ikuro Nakamura, Kusuki Nishioka, Toshihiro Nakajima

Scientific Reports 6: Article number: 3694310.1038/srep36943; published online: 11 11 2016; updated: 05 11 2018

The Publisher is retracting this Article because the experimental approach does not support the objectives of the study. The study was designed to elucidate the maximum implication of human papilloma virus (HPV) vaccine (Gardasil) in the central nervous system. However, the co-administration of pertussis toxin with high-levels of HPV vaccine is not an appropriate approach to determine neurological damage from HPV vaccine alone. The Authors do not agree with the retraction.

